# A Survey of Medical Oncology Training in Australian Medical Schools: Pilot Study

**DOI:** 10.2196/mededu.7903

**Published:** 2017-12-12

**Authors:** Mathew George, Hiren Mandaliya, Amy Prawira

**Affiliations:** ^1^ Medical Oncology North West Cancer Centre Tamworth Australia

**Keywords:** medical, oncology, training, Australia

## Abstract

**Background:**

Oncology is a rapidly evolving field with continuous advancements in the diagnosis and treatment of cancer. Therefore, it is important that medical students are provided with the knowledge and experience required to care for oncology patients and enable them to diagnose and manage toxicities of novel therapeutic agents.

**Objective:**

This study was performed to understand the medical students’ perspective of the oncology education provided in universities across Australia and identify areas of education that could potentially be modified or improved to ultimately attract more students to a career in oncology.

**Methods:**

This pilot cross-sectional study consisted of an 18-question survey that was submitted online to medical students in their final year and interns rotating to the Tamworth Hospital.

**Results:**

The survey was completed by 94 fifth-year medical students and interns. Oncology was taught both theoretically and clinically for 68% (63/93) of participants, and 48% (44/92) had an exclusive oncology rotation. Both theoretical and clinical oncology assessments were conducted for only 21% (19/92) of participants. Overall, 42% (38/91) of participants were satisfied with their oncology education, and 78% (40/51) were dissatisfied with the number of oncology teaching hours. The importance of a career in oncology was rated as low by 46% (41/90) of participants.

**Conclusions:**

This pilot study indicates that there are potential areas to improve oncology teaching in Australian universities. The majority of surveyed students were dissatisfied with the number of teaching hours they receive in oncology. More global assessment of students and/or interns from other Australian institutes may yield further useful information.

## Introduction

Oncology is a rapidly advancing field with novel treatment options and methods of diagnosis being continuously developed for many types of cancer. These put a significant burden on junior and senior clinicians as they are required to maintain an up-to-date understanding of novel treatments and modes of diagnosis to provide patients with a high standard of care. To suitably prepare junior doctors, a tertiary education is required that provides them with the capability to not only diagnose and treat patients but also to detect, as early as possible, the symptoms of the acute toxicities associated with both novel and conventional treatments. Furthermore, clinicians need the skill required to continuously incorporate the latest developments in the field into their repertoire. Currently, there is no standard method to ensure that the oncology curriculum in medical schools is of a quality that sufficiently prepares medical students to care for oncology patients.

Worldwide, there is considerable variation in the content and structure of the oncology education taught in medical schools. For many universities, medical oncology rotations are often not mandatory [1,2]. In 2007, a survey of recently graduated interns from the United Kingdom revealed only 40% of participants felt prepared to diagnose cancer, 15% felt that they had sufficient knowledge of radiotherapy and chemotherapy, and 11% felt prepared to treat an oncological emergency [3]. Similar results were obtained from a 2013 survey of 82 interns in India, with only 32% of interns being aware of the role of radiotherapy, only 37.5% of interns being aware of the role of chemotherapy, and only 12.5% of interns being confident caring for terminal and late stage patients [4].

There is evidence that oncology is underrepresented in the curricula of Australian medical schools, and concern has been raised regarding the extent of the exposure of students to oncology [5]. McRae et al [5] compared the cancer knowledge and skills of interns graduating from graduate medical program courses with those from non–general medical program courses and also compared the cancer knowledge and skills of interns in 2001 with those who completed a similar survey in 1990 [6] and concluded that graduates from 2001 had less exposure to specific cancers such as melanoma, rectal cancer, and mouth cancer than those who trained in 1991. The study was guided by the Australian Cancer Society’s Ideal Oncology Curriculum for Medical Schools, which was established in 1999 and has been regularly updated, with the last revision in 2014. Findings from McRae et al [5] suggested that the oncology education provided to medical students could be structured more effectively to provide students with a greater appreciation of the field, which may generate more interest in oncology as a future career. Hence, we believed another study to understand the knowledge and skills of medical students and interns for medical oncology was in order. The aim of this pilot cross-sectional study was to gain an understanding of medical students’ perspectives of the oncology education provided in universities across Australia and identify potential areas of the tertiary education that could be modified or improved to ultimately attract more students to a career in oncology.

## Methods

### Study Design

This pilot cross-sectional study consisted of an online questionnaire developed by the investigators (see Multimedia Appendix 1). The survey was completed between August 2013 and August 2015 and consisted of 18 questions. Participation was offered to all fifth-year medical students and interns rotating through North West Cancer Centre and Tamworth Rural Referral Hospital (Tamworth, New South Wales, Australia). The questions were separated into 5 categories: institutions, exposure to oncology, oncology curriculum and teaching, students’ perceptions of the curriculum, and interest in pursuing oncology as a career. This study was approved by the human research ethics committee at the University of New England, New South Wales, Australia. All participants provided written informed consent.

### Participants

The study population consisted of medical students in their final year of study or first-year postgraduate students (interns) from Australian medical schools. An open invitation was submitted to the students and interns who rotated through North West Cancer Centre and Tamworth Rural Referral Hospital during the survey period.

### Statistical Analysis

Statistical analysis was performed using SAS version 9.4 (SAS Institute Inc). Data were presented in the form of numerical values, transformed into percentile values, classified into 3 categories of variables with 2 sets of values, and inference obtained on direct or inverse proportionality of the variables.

## Results

### Participant Population

A total of 94 medical students or interns were recruited and completed the questionnaire. The universities represented are displayed in Table 1.

### Exposure to Oncology During Medical School

When asked which year of medical school participants were first introduced to oncology, the majority of participants who responded to the question reported that it was introduced to them in their fifth year of study (53/93, 57%) (Table 2). Eleven, 10, 8, and 9 participants reported that they were introduced to oncology in first, second, third, and fourth years, respectively. One participant was not introduced to oncology until their sixth year, and one reported to have never received oncology education in medical school.

**Table 1:** Participating universities.

University	Number of participants (N=94)
University of Newcastle	37
University of New England	18
University of New South Wales	13
University of Wollongong	3
Sydney University	7
Other	12
Did not respond	4

**Table 2:** Year of introduction to oncology.

Year of medical school	Number of respondents N=93, n (%)
One	11 (12)
Two	10 (11)
Three	8 (9)
Four	9 (10)
Five	53 (57)
Six	1 (1)
Never	1 (1)

### Oncology Education

Participants were asked whether they were taught oncology in theory only, whether they were taught during clinical rotations only, or if both teaching methods were employed. Out of the 93 responding participants, 3 (3%) were taught the theory only, 25 (27%) were taught during clinical rotations only, and 63 (68%) were taught with both methods. Two (2%) participants stated that they were not taught medical oncology or did not respond. Most participants received between 1 and 5 weeks of education (78/93, 84%), 11 (12%) received 5 to 10 weeks, and no participants received 10 weeks or more.

Rotations were not always exclusively dedicated to a single specialty and may have been used to teach multiple topics. Out of 92 responding participants, 44 (48%) had an exclusive oncology rotation and 44 (48%) had oncology combined with another specialty (Table 3). Four participants were unsure. The oncology rotation was mandatory for 75% (70/92) of participants and elective for 17% (16/92) of participants; 9% (8/92) of participants were unsure.

When asked if there was knowledge testing in oncology, 49% (45/92) of participants reported that they were not assessed, while 21% (19/92) of participants reported undergoing both theoretical and clinical examinations; 24% (22/92) had only written assessment, and 7% (6/92) had only clinical examination.

To determine why participants may have limited oncology education, participants were asked if any medical oncologists were involved in teaching at their university. Half (45/94, 50%) stated that medical oncologists were involved in teaching at their university, 9% (8/94) reported that they did not receive any teaching from a medical oncologist, 41% (37/94) were unsure, and 4 did not answer the question. These data suggest that half of all medical students either did not have access to or were not aware that they had access to a teaching medical oncologist at their university.

### Student Assessment of Their Oncology Education

When participants were asked to grade the quality of their oncology education as either satisfactory, average, or unsatisfactory, 42% (38/91) participants rated their oncology education as satisfactory, 48% (44/91) rated this at average, and 10% (9/91) reported that it was unsatisfactory. When participants were asked to indicate reasons why they were dissatisfied with their oncology education, 78% (40/51) of responding participants indicated that they were dissatisfied with the limited number of teaching hours, 65% (33/51) of participants attributed this to a lack of clinical exposure, 29% (15/51) of participants believed there was a lack of consultant training sessions, and 26% (13/51) stated that they had limited resources.

Conversely, of the participants that identified aspects of their medical oncology training that they found satisfactory, 78% (57/73) of participants attributed this to oncology consultant teaching, 66% (48/73) of responders attributed this to adequate teaching exposure, 48% (35/73) to adequate teaching hours, and 38% (28/73) to adequate access to resources.

When asked which were the medical oncology topics the participants felt needed more attention in medical school, 49% (44/89) suggested clinical application, while 17% (15/89) recommended that more attention be given to treatment approaches (Table 4).

### Oncology as a Career

When participants were asked to rate their view of the importance of a future career in medical oncology as either low or high, 54% (49/90) rated the importance as high and 46% (41/90) reported the importance as low.

**Table 3:** Methods by which students were taught oncology.

Teaching method	Number of respondents N=93, n (%)
Theory only	3 (3)
Clinical rotations only	25 (27)
Both methods	63 (68)
Not taught	2 (2)

**Table 4:** Oncology topics medical students believed should be given more attention.

Areas of oncology	Number of respondents N=89, n (%)
Molecular biology	2 (2)
Pathophysiology	7 (6)
Pathology	0 (0)
Clinical applications	44 (49)
Diagnostic investigations	10 (11)
Treatment approaches	15 (17)
Psychosocial aspects	4 (5)
Other	7 (8)

The 2 main reasons why the medical students and interns would not choose medical oncology as a career were lack of sufficient understanding or awareness of the topic (47/72, 65%) and lack of sufficient exposure at the undergraduate level (25/72, 35%). No participant stated that they would not specialize in oncology due to a lack of career prospects.

## Discussion

### Principal Findings

This cross-sectional pilot study investigating medical students’ perspectives on oncology education highlights potentially significant differences in teaching methods and students’ understanding and exposure to oncology across Australian universities.

The supply of medical oncologists is currently insufficient for the incidence of cancer in Australia, and the demand for oncologists is expected to increase as the aging population continues to develop [7]. Therefore, it is important that a sufficient number of medical students choose to specialize in oncology. However, in this study, 46% of medical students graded the importance of a career in medical oncology as low.

Previous studies indicate that the quality and quantity of the education provided in a subject is an important factor in student decisions to specialize in that field [8,9]. A study completed by French oncology residents found that exposure to oncology as a medical student was a factor involved in 83% of student decisions to choose oncology as a specialty [8]. Furthermore, a survey completed by 488 participants from 14 medical schools in the United Kingdom found that students were more likely to choose urology as a specialty if they had more hours of urology teaching, if they attended urology theater sessions, and if they had confidence in performing urological procedures [10]. This may in part explain the lack of interest in oncology by Australian medical students, as only 68% were taught oncology theoretically and clinically, 79% were dissatisfied with the number of teaching hours, and 68% were dissatisfied with their level of clinical exposure. A study that found that Australian medical interns in 2006 had less opportunity to examine cancer patients than interns did in 1990 [5,6]. These data raise a question whether the oncology education provided by Australian universities is of sufficient quality and quantity to gain the interest of students and to make them feel confident that they have the knowledge and skills required to enter the specialty. Our participants were not the true representation of nationwide universities and their medical oncology teaching program. We think structured and collaborative future studies in this direction would be essential to address these important aspects.

The development of a standardized curriculum to improve student education in the rapidly changing field of oncology is crucial to ensuring that medical graduates are well equipped to care for oncology patients. Therefore, we propose the development of a centralized body to standardize the oncology curriculum across Australian medical schools by updating the Ideal Oncology Curriculum or starting a new process altogether, thereby ensuring a high-quality oncology education for all medical students.

Results from this pilot study suggest that an exclusive oncology rotation may be of value in improving students’ confidence and interest in the field. Indeed, this result is supported by another survey where a brief 2-week rotation was found to have significant value in improving student’s confidence to care for patients in an oncology clinic [11]. The oncology curriculum could also be improved by making it mandatory for all medical students to complete an exclusive oncology rotation. This is evident as students who complete exclusive rotations are more likely to choose to specialize in that field [11-13]. A survey completed by 36 medical students before and after an oncology clinical rotation found that students were more confident in an oncology clinic after the rotation [11]. In our study, only 43% of the participants completed an exclusive oncology rotation, and 64% stated that they would not specialize in oncology because they lacked a sufficient understanding of the field. Therefore, by making it mandatory for all medical students to complete an exclusive oncology rotation, students are more likely to gain confidence and subsequently choose oncology as a specialty.

### Limitations

The limitations of this pilot study include the low number of participants recruited and the enrollment of only medical students and interns rotating through a rural Australian center. These factors limit its generalizability and the ability to draw meaningful conclusions. Furthermore, some participants were medical oncology interns on oncology rotations, who may have been biased because of their oncology experience. The lack of participant demographics, while ensuring anonymity and encouraging participants to speak freely, is also a weakness.

### Conclusions

Nevertheless, this small pilot study indicates that this group of Australian medical students is receiving education in medical oncology that could be improved. The lack of satisfaction with the quality of the education may be influencing the low numbers of students choosing to specialize in medical oncology. The area identified as requiring additional emphasis in this survey is the clinical application. A more detailed and broader survey may further delineate potential areas of priority in improving oncology education in tertiary institutions.

## Appendix

Multimedia Appendix 1 Online survey questions.
