# Supplementary material for: A Survey of Medical Oncology Training in Australian Medical Schools: Pilot Study
Source: JMIR Med Educ. 2017 Dec 12;3(2):e23. doi: 10.2196/mededu.7903 (PMC5743919; doi:10.2196/mededu.7903)
Supplement: Multimedia Appendix 1 [file mededu_v3i2e23_app1.pdf]

Multimedia Appendix 1

Survey questions

1) In which University are you studied?
2) What was your clinical school?
3) In which year you were introduced to medical oncology?
   A. 1st year
   B. 2nd year
   C. 3rd year
   D. 4th year
   E. 5th year
   F. 6th year
   G. Never
4) How was medical oncology taught?
   A. Theory only
   B. Clinical rotation
   C. Both of above
   D. Never
5) How many weeks were you assigned to medical oncology teaching?
   A. 1-5 weeks
   B. 5-10 weeks
   C. 10 or more weeks
   D. None
6) Kindly specify the number of weeks of medical oncology teaching.
7) Is medical oncology a rotation on its own?
   A. Yes
   B. Combined with another
   C. Unsure
8) How were you assessed in medical oncology?
   A. Theory only
   B. Clinical examination
   C. Both of above
   D. Never
9) Is a rotation in medical oncology compulsory?
   A. Mandatory
   B. Elective
   C. Unsure
10) How would you rate the quality of medical oncology teaching in your medical curriculum?
   A. Satisfactory
   B. Average
   C. Unsatisfactory
11) Reasons of unsatisfactory training in medical oncology
   A. Lack of consultant teaching sessions
   B. Limited hours of teaching
   C. Lack of clinical exposure
   D. Limited resources
12) Reasons for satisfactory training in medical oncology

A. Consultant teaching
B. Adequate teaching hours
C. Adequate clinical exposure
D. Adequate resources

13) Are there any medical oncologists who teach at your university?
   A. Yes
   B. No
   C. Unsure
14) In your opinion, medical oncology should be taught as -
   A. A subject on its own
   B. In combination with other subjects/specialities
15) In which of the following topics should more emphasis be given?
   A. Molecular biology
   B. Pathophysiology
   C. Pathology
   D. Clinical applications
   E. Diagnostic investigations
   F. Treatment approaches
   G. Psychosocial aspects of medicine
   H. Others (please specify)
16) How would you rate the importance of medical oncology as a future career?
   A. High
   B. Low
17) What is your field of preference for future training?
   A. Adult medicine (including medical oncology)
   B. Surgery
   C. Radiation oncology
   D. Radiology
   E. Pathology
   F. Other
18) In your opinion, what would be the reason for not choosing medical oncology as a career?
   A. Lack of exposure at an undergraduate level
   B. Lack of awareness
